# Graves' Disease Presenting as Urticaria

**DOI:** 10.7759/cureus.12134

**Published:** 2020-12-17

**Authors:** Mohammed A Al Rushud, Mohammed Gaffar Mohammed, Abdulrahman Murshid, Saud Almuammar, Abdullah M AlHossan

**Affiliations:** 1 Internal Medicine, Alfaisal University, Riyadh, SAU; 2 Internal Medicine, Dr. Sulaiman Al-Habib Hospital - AL Sweidi Branch, Riyadh, SAU; 3 Internal Medicine, Alfaisal University College of Medicine, Riyadh, SAU; 4 Surgery, Alfaisal University College of Medicine, Riyadh, SAU

**Keywords:** graves-basedow, urticaria, allergic reaction

## Abstract

Graves’ disease (GD) is an autoimmune disease that is characterized by the presence of antibodies targeting the thyroid gland. Commonly, the disease presents with symptoms of thyrotoxicosis such as sweating, tremors, and weight loss; less frequently, patients with GD might also have urticaria. Urticaria is clinically defined as the presence of wheals, angioedema, or both. While its pathophysiology is not completely understood, urticaria is believed to be an immune-mediated response activating mast cells, leading to the release of histamine and cytokines. Although the association between urticaria and GD is well established, it is uncommon for GD patients to present with urticaria as their sole complaint. In this paper, we present a case of a previously healthy patient who originally presented to the dermatology clinic with urticaria alone; however, she developed thyrotoxic symptoms soon after. Furthermore, her treatment was complicated by developing an allergic reaction to carbimazole.

## Introduction

Graves’ disease (GD) is an autoimmune disorder that is characterized by the presence of anti-thyroid stimulating hormone (TSH) receptor antibodies causing thyrotoxicosis [1]. It is well recognized as the most common cause of hyperthyroidism in the world [2]. The signs and symptoms of GD vary widely but one of the least common and less understood of these symptoms is urticaria. The first association between thyroid disease and urticaria was made all the way back in 1907 by Ravitch at the 5th International Dermatological Congress. At the same congress, Ravitch also discussed the effects of treating thyroid disease on urticaria [1]. 

The earliest reports of urticarial manifestations in patients with hyperthyroidism actually predate those of autoimmune thyroiditis. In subsequent years, the link between thyroid disease and urticaria was analyzed by multiple studies [3]. The relationship between Graves’ and urticaria is complex to say the least and its nature is not yet well understood. However, a common autoimmune etiology has been suggested all the way back in 1972 in which Issacs described four patients suffering from thyrotoxicosis and chronic urticaria that was resolved by normalizing the thyroid function [1]. In this paper, we discuss a case of GD that presents as chronic urticaria and we review the literature about the relationship between these two pathological conditions.

## Case presentation

A previously medically free 48-year-old female was referred from the dermatology clinic with a complaint of generalized pruritic urticaria refractory to conventional treatment of non-sedating antihistamines (allerfin 4 mg + XYZAL 5 mg) and topical corticosteroids (mometasone 0.1% QD). The pruritic urticaria started three days prior to presentation; however, she has been having similar episodes over the past few months but never as severe as her current presentation. General laboratory investigations were performed and the results were significant for undetectable TSH levels. Given these results, the patient was referred to Internal Medicine where further history and examinations were performed. The patient revealed to have been suffering from moderate cardiac palpitations, weight loss, heat intolerance, polyphagia, oligomenorrhea and diarrhea over the past four months. The patient had originally dismissed these symptoms as perimenopausal symptoms. On physical examination, the patient was found to have urticarial rash, staring look, fine tremors and a stage 3 goiter. However, all these thyrotoxic signs and symptoms were mild in this patient and she was notably negative for eye symptoms, lid lag and lid retraction.

**Figure 1:**
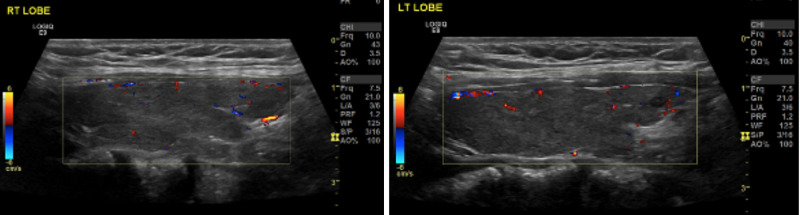
Duplex Doppler ultrasound of left and right lobes, showing a bilateral decrease in vascularity.

The patient was started on propranolol 20 mg BID and complete thyroid function tests with thyroid antibodies were performed alongside an ultrasound of her thyroid and cervical lymph nodes. The ultrasound showed a mildly enlarged thyroid, measuring about 12 cc in size, with heterogeneous hypoechoic pattern and decreased vascularity that is suggestive of post-inflammatory thyroiditis. There were no suspicious focal thyroid lesions and cervical lymph nodes looked benign bilaterally (Figure 1).

Lab results came back typical for primary hyperthyroidism with high free T3 and T4 but low TSH. Also, TSH receptor antibodies and anti-thyroglobulin antibodies came back positive which confirmed the diagnosis of GD. Notably, the patient IgE titer was also elevated.

After confirming the diagnosis, the patient was started on carbimazole 10 mg BID and was booked for follow-up in six weeks. During the first week of treatment, the patient developed a shortness of breath and a severe pruritic rash. She discontinued the medication by herself and her symptoms improved over the following week. Once the patient returned to baseline, she restarted carbimazole 10 mg QD, yet again she developed a shortness of breath and a severe pruritic rash. She discontinued the medication and presented to the clinic the following day. In the clinic, her hyperthyroidism was found to have gotten worse. The patient has also developed lid lag and lid retraction which were absent on the original presentation. Blood was sent for thyroid function tests (TFTs) which came back even higher than her original lab results. Her gamma-glutamyl transferase (GGT) was also elevated suggesting some hepatotoxicity from carbimazole. The patient was switched to propylthiouracil (PTU) 50 mg TID and was evaluated for possible radioactive iodine therapy.

After six weeks on PTU, the patient was reevaluated and she showed a marked improvement in her symptoms. Even her initial complaint of pruritic urticaria is now completely controlled on a low dose of antihistamines; however, she still had some lid lag and fine tremors. Laboratory studies also showed a marked improvement in TFT and GGT which normalised after discontinuing Carbimazole. The patient was followed up every six weeks and showed continuous improvements; by her sixth month of treatment, she was found to be clinically euthyroid with normal free T3 and T4 levels.

## Discussion

GD is an autoimmune disease that is triggered by a mixture of polygenetic and environmental factors. It is the most common cause of hyperthyroidism worldwide accounting for 60% to 80% of hyperthyroid cases and is more common in females than males with a reported lifetime risk in women and men of 3% and 0.5%, respectively [2,4]. The reported numbers are not that different in Saudi Arabia with a female:male ratio of 2.9:1 and a mean age of 32 +/-0.9 years [5].

The hallmark of GD is the presence of thyroid-stimulating auto-immunoglobulins that bind to and activate TSH receptors, causing hyperstimulation of the thyroid gland, and symptoms of hyperthyroidism. Given the systemic nature of GD, these symptoms may affect any system and usually they include heat intolerance, sweating, fatigue, weight loss, palpitation, hyper defecation, and tremors. On physical examination for GD, clinicians should examine for ophthalmopathy, including eyelid retraction, proptosis, and periorbital edema. Another sign of GD is thyroid dermopathy which causes thickening of the skin mainly over the tibia; however, it is a rare finding [4,6].

To diagnose GD, a thorough clinical evaluation is indicated to uncover any signs or symptoms of the disease. Once GD is suspected, laboratory tests should be carried out for definitive diagnosis. Initially, a thyroid function test must be performed to establish hyperthyroidism. If the TSH is suppressed, we need to establish the levels of free T4 and free T3 as well. To distinguish GD from other causes of hyperthyroidism we measure the level of TSH receptor antibody (TRAb). The measurement of TRAb with third-generation assay has a sensitivity of 97% and specificity of 99% for GD [4,7].

Treatment of GD relies on normalising thyroid hormone levels and controlling the symptoms of hyperthyroidism. Symptomatic control is usually accomplished by beta blockers while thyroid hormone levels are treated with antithyroid medications, radioactive iodine therapy or surgical thyroidectomy. All of these options for hyperthyroidism are established to be effective and treatment plans that only differ in their use according to patients' presentation. The main antithyroid medications used in hyperthyroidism are thionamides which include Propylthiouracil and Methimazole. They work by inhibiting thyroid peroxidase which mediates iodination of thyroglobulin. Side effect profile of these medications include: hepatotoxicity, neutropenia, and hypersensitivity reactions. A reported incidence of 3%-6 % of the patients had hypersensitivity reactions, which included pruritus, and rash [4].

Urticaria, specifically chronic idiopathic urticaria (CIU) as in our case, is a relatively common presentation with an estimated population prevalence that ranges from 0.5% to 5%. It is defined as the development of wheals, angioedema or both for more than a six-week period [8]. The pathophysiology of CIU is not fully understood, but the main factor is triggering an immune response and thereby releasing histamine and cytokines. The findings of autoantibodies against the alpha subunit of the high-affinity IgE receptor and against IgE itself in about one-third of patients with CIU have suggested an autoimmune etiology to the disease [9]

The diagnosis of CIU is somewhat difficult. Initially, the presence of wheals and angioedema alone would point towards CIU; however, If the patient presents with respiratory, gastrointestinal or constitutional signs or symptoms, other diagnoses such as allergic reactions should be ruled out first. Once CIU is diagnosed, its treatment relies on targeting the H1 receptors, using a nonsedating antihistamines. Also, exposure to any triggers that exacerbate the urticaria must be avoided [10]. Furthermore, medications other than antihistamines, namely Leukotriene antagonists, systemic steroids and monoclonal antibodies, are now more commonly used but they still remain inferior to antihistamines as a first-line therapy. 

There is an association between CIU and multiple autoimmune diseases, of which thyroid autoimmune disease is the most common [11]. Autoimmune thyroiditis and the presence of anti-thyroid antibodies have been reported in patients suffering CIU with a frequency of 6.5% to 57% [12]. In a 2017 systematic review by Kolkhir et al. [1], the assessment of 68 studies found an increase in IgG antithyroid peroxidase (IgG-anti-TPO) and IgG anti-thyroglobulin (IgG-anti-TG) in patients with CIU. The same review also looked at six independent studies and found levels of IgG anti-TSH receptor (IgG anti-TSHr) to be elevated in patients with CIU. Although most of these studies suggested that the findings of such antibodies were part of an independent, parallel disease process, a recent study proposed a different idea [13]. Altrichter et al. showed that a sizable subgroup of patients with CIU had increased IgE antibodies against thyroid peroxidase (TPO) [14]. These anti-TPO IgE antibodies, when bound to the surface of mast cells, could cause activation and degranulation of mast cells, thus playing an active role in the pathogenesis of CIU [13,14]. In the end, while suspected, a common pathogenesis between CIU and autoimmune thyroid disease is difficult to prove. Largely, this is due to the multitude of reports of associations between autoimmune diseases, making an overall susceptibility of a patient with an autoimmune disease, such as GD or CIU, to other autoimmune diseases much more likely [13]. 

In their assessment of 154 patients with CIU, Small and Lerman found six patients with autoimmune hyperthyroidism [1]. Another study by Collet et al. [13] found eight among 45 patients with CIU suffering from autoimmune thyroid disease; of those only one was suffering from GD. Gaig et al. [13] measured serum Ab-Tg and Ab-TPO in 170 patients with CIU, and found that 45 of them (14.7%) had at least one antithyroid antibody; of the 45, only 2 had GD. In a more recent study by Confino-Cohen et al. The prevalence of autoimmune thyroid disease was evaluated in 12, 778 patients with CIU, compared to a control group of 10, 714 [15]. Antithyroid antibodies (TPO-Ab and TG-Ab) were found to be significantly higher in patients with CIU than in control groups, and 10% of patients with CIU had hypothyroidism compared with 0.6% of controls. The prevalence of hyperthyroidism, although less common than hypothyroidism, was also significantly higher in patients with CIU than in the control group (2.6% and 0.09% respectively). Overall, these studies concluded that the association between CIU with GD is present but to a lesser extent than that of CIU and autoimmune thyroiditis [1].

Another well-documented association is that of GD and allergic diseases. This has been supported by the elevation in serum levels of IgE in one-third of patients with GD, but not in patients with Hashimoto thyroiditis [16]. GD patients with elevated IgE also showed low rate of remission when treated with antithyroid drugs and high rate relapse [17]. Furthermore, there have been reports suggesting an improvement in CIU after therapeutic correction of hyperthyroidism [13]. Recently, Bansal and Hayman [18] described two patients with CIU that were poorly responsive to antihistamines and corticosteroids, who then developed symptoms of hyperthyroidism within 6 months of the onset of urticaria. In both patients, treatment of the hyperthyroidism with carbimazole and normalization of their thyroid function quickly improved their urticaria [1]. A possible mechanism might be because antithyroid drugs improve hyperthyroidism not only by decreasing the synthesis of thyroid hormones, but also by decreasing anti-thyroid antibodies probably due to immunosuppressive effects. In the same manner, this immunosuppressive effect may influence CIU of autoimmune origin [19]. However, due to the scarcity of these reports, it is difficult to ascertain whether the improvement in urticarial symptoms is due to the antithyroid drugs or whether it is a natural improvement of the disease itself [1].

## Conclusions

The association between GD and urticaria is well established and it is recommended to evaluate patients with urticaria for thyroid disease even when asymptomatic. However, there is a need for more research to better understand the nature of the relationship between GD and urticaria. Also, more research is needed to establish the time course for developing thyroid symptoms in urticarial patients. Finally, the relationship between Graves’ associated with urticaria and hypersensitivity reactions should be better studied for its important implications on clinical practice and patient education.
